# Increase in the Prevalence of Resistance Determinants to Trimethoprim/Sulfamethoxazole in Clinical *Stenotrophomonas maltophilia* Isolates in China

**DOI:** 10.1371/journal.pone.0157693

**Published:** 2016-06-16

**Authors:** Li-Fen Hu, Guo-Sheng Chen, Qin-Xiang Kong, Li-Ping Gao, Xi Chen, Ying Ye, Jia-Bin Li

**Affiliations:** 1 Department of Infectious Diseases, The First Affiliated Hospital of Anhui Medical University, Hefei, Anhui, China; 2 Anhui Center for Surveillance of Bacterial Resistance, Hefei, Anhui, China; 3 Department of Infectious Diseases, The Chaohu Affiliated Hospital of Anhui Medical University, Hefei, Anhui, China; Universidad Nacional de la Plata, ARGENTINA

## Abstract

**Aims:**

This study was carried to reveal the genetic mechanisms of trimethoprim/sulfamethoxazole (SXT) resistance.

**Methods:**

Among 300 clinical *Stenotrophomonas maltophilia* isolates from China, resistance determinants such as *sul* and *dfrA* genes, integrons and transposase were examined using PCR, DNA sequencing and thermal asymmetric interlaced PCR (TAIL-PCR). Data were analyzed using SPSS 20.0.

**Results:**

Of the 300 isolates, 116 (38.7%) were resistant to SXT. An alarming trend of increased resistance to SXT were found over the 10-year period. The positive rates of *sul* and class 1 integrase (*intI1*) increased gradually with the development of SXT resistance over the 10-year period. Multiple logistic regression analyses indicated that the genes of *qacEΔ1-sul1* (81% *vs* 46.2%, *p* = 0.000), *sul2* (50.9% *vs* 9.8%, *p* = 0.000), *intI1* (83.6% *vs* 65.8%, *p* = 0.000), *dfrA12* (25% *vs* 3.3%, *p* = 0.000), *dfrA17* (15.5% *vs* 3.8%, *p* = 0.000) and *dfrA27* (4.3% *vs* 1.6%, *p* = 0.01) were more prevalent in SXT-resistant isolates than SXT-susceptible isolates except *dfrA1*(*p* = 0.83) and *dfrA5*(*p* = 0.18). Sequencing data revealed 12 types of resistance gene cassettes (*aar-3*-*dfrA27*, *dfrA12*–*aadA2*, *dfrA17*–*aadA5*, *cmlA1*, *aacA4*, *aadA5*, *arr-3-aacA4*, *aadA1*, *aadB–aadA4*, *aacA4*–*catB8*–*aadA1*, *aadB*–*aac(6****′****)-II*–*bla*_CARB-8_ and *aac(6****′****)-II*–*bla*_*CARB-8*_) located in the class 1 integron in 163 isolates (87% SXT-resistant *vs* 33.7% SXT-susceptible isolates, *p* = 0.000). A novel finding was the *aar-3*-*dfrA27* (KC748137) gene cassette. The gene of *sul2* linked to transposase in 50 SXT- resistant and 7 SXT- susceptible isolates was detected by TAIL-PCR.

**Conclusions:**

The findings demonstrated a higher prevalence of *sul*, *dfrA*, *intI1* and resistance gene cassettes in class 1 integron in SXT-resistant clinical *S*. *maltophilia* isolates in China. The *sul1* and *dfrA* genes located in integrons and the *sul2* linked to transposase may imply wide and rapid dissemination of resistance gene in bacteria.

## Introduction

*S*. *maltophilia*, a non-fermentative gram-negative bacterium, is generally regarded as an important opportunistic pathogen, especially in immunocompromised patients with underlying disease, and can cause a number of clinical syndromes, such as bacteraemia, sepsis, pneumonia, meningitis, endocarditis, septic arthritis, urinary infections, and endophthalmitis [1–3].

*S*. *maltophilia* has been recognized as one of the leading multidrug resistant organisms in hospital settings due to its resistance to a broad array of antimicrobial agents afforded by the existence of intrinsic and acquired resistance mechanisms [4]. Trimethoprim/sulfamethoxazole (SXT) is traditionally recommended as the first choice against *S*. *maltophilia* infections; however, increasing resistance to SXT has complicated the treatment. Resistance determinants such as *sul* and *dfrA* genes, class 1 integrons and mobile genetic elements have been reported to contribute to SXT resistance [5–6], but these determinants were sometimes also detected in SXT- susceptible *S*. *maltophilia* isolates. To our knowledge, there is no detailed study describing the association between SXT resistance and resistance genes in a large collection by multivariate statistics.

In this study, the in vitro susceptibility of SXT against 300 clinical *S*. *maltophilia* isolates were examined to reveal the evolution of SXT resistance, SXT resistance determinants such as the *sul* and *dfrA* genes and resistance gene cassettes in integrons were determined, and their contribution to SXT resistance were analysed by multivariate statistics.

## Materials and Methods

### Bacterial isolates

A total of 300 clinical *S*. *maltophilia* isolates were collected from different patients after 72 h of hospitalization in 25 hospitals in Anhui, China in 2005–2014. The 300 isolates were comprised of 236 (78.67%) from sputum specimens, 26 from secretion (8.67%), 14 from urine (4.67%), 11 from blood (3.67%), 10 from drainage (3.33%), and 3 from cerebrospinal fluid (1.0%). All *S*. *maltophilia* isolates were identified using the MicroscanWalkaway-40 System (Dade Behring, Deerfield, IL, USA) and were re-identified via 16S rRNA sequencing with specific primers in Table 1. Quality control strain *Pseudomonas aeruginosa* ATCC 27853, *Escherichia coli* ATCC 35218, and *Escherichia coli* ATCC 25922 were stored at the Anhui Center for Surveillance of Bacterial Resistance.

**Table 1:** Oligonucleotide primers used in this study.

primers	Sequences(5'-3')	Target	Reference
*intI1-R*	CAGTGGACATAAGCCTGTTC	class1 integrase	6
*intI1-F*	CCCGAGGCATAGACTGTA		
*In1-R*	AAGCAGACTTGACCTGA	class 1 integron	8
*In1-F*	GGCATCCAAGCAGCAAG		
*hep74*	CGGGATCCCGGACGGCATGCAC	class 2 integron	9
*hep51*	GATGCCATCGCAAGTACGAG		
*int3*-F	AGTGGGTGGCGAATGAGTG	class 3 integranse	10
*int3-F*	TGTTCTTGTATCGGCAGGTG		
*sul1*-R	ATTCAGAATGCCGAACACCG	*sul1*	6
*sul1*-F	TAGCGAGGGCTTTACTAAGC		
*sul2*-R	GAAGCGCAGCCGCAATTCAT	*sul2*	6
*sul2*-F	CCTGTTTCGTCCGACACAGA		
*dfrA1*-R	TTGTGAAACTATCACTAATGGTAG	*dfrA1*	6
*dfrA1*-F	CTTGTTAACCCTTTTGCCAGA		
*dfrA5*-R	TCCACACATACCCTGGTCCG	*dfrA5*	6
*dfrA5*-F	ATCGTCGATATATGGAGCGTA		
*dfrA12*-R	ATGAACTCGGAATCAGTACGC	*dfrA12*	6
*dfrA12*-F	TTAGCCGTTTCGACGCGCAT		
*dfrA13*-R	GAAACTATCACTAATGGCAGC	*dfrA13*	6
*dfrA13*-F	CTCATCTGCTGGCTATCTCA		
*dfrA17*-R	TTGAAAATATTATTGATTTCTGCAGTG	*dfrA17*	6
*dfrA17*-F	GTTAGCCTTTTTTCCAAATCTGGTATG		
*dfrA27*-R	TAAAGCAATAACTTACAATC	*dfrA27*	This study
*dfrA27*-F	AAGAGTCTGATCGCCCATGCCG		
*sul2*-SP1	AAAGAACGCCGCAATGTGATCC	3’flanking nucleotide of *sul2*	This study
*sul2*-SP2	GGATAGAACGCAGCGTCTGGAAAA		
*sul2*-SP3	CATCTGCCTTGAGCGCGTCC		
*sul2-*SP4	GTGGGAATGAGTTGGAAGAA	5’flanking nucleotide of *sul2*	This study
*sul2*-SP5	GTTGCCGTTGTGGGTGCTGT		
*sul2*-SP6	TGCGCTATCGTGGGGGAATG		
16s RNA-R	ACGGCAGCACAGAAGAGC	16s RNA	This study
16s RNA-F	AGTTCGCATCGTTTAGGG		

SP, special primer; F, forward; R, reverse.

### Antimicrobial susceptibility testing

Minimal inhibitory concentration (MIC) of trimethoprim/sulfamethoxazole against each isolate was determined by the agar dilution method according to the Clinical and Laboratory Standards Institute (CLSI) guidelines [7]. Quality control strains were included in each batch of antimicrobial testing to ensure the accuracy of the results. Isolates with MIC to trimethoprim/sulfamethoxazole < 2/38 mg/L were defined as susceptible isolates and resistant isolates with MIC to trimethoprim/sulfamethoxazole > 4/76 mg/L suggested by the CLSI [7].

### Polymerase chain reaction (PCR)

The presence of class 1, 2 and 3 integrons as well as *qacEΔ1-sul1*, *sul2* and *dfrA1*, *dfrA5*, *dfrA12*, *dfrA17* and *dfrA27* genes in each strain were assessed using the primers shown in Table 1[6, 8–10] using previously described conditions [6] using PCR (Biometra, Germany). Gene cassettes embedded within the class 1 integrons were also determined using the primers specific to the 5'conserved segment (CS) and the 3' CS listed in Table 1.

### Thermal asymmetric interlaced PCR (TAIL-PCR)

For TAIL-PCR, three nested sequence-specific primers were utilized in consecutive reactions together with arbitrary degenerate (AD) primers to enhance the amplification efficiency of specific products [11]. Three specific primers (Table 1) and four AD primers (AD1, AD2, AD3 and AD4) from the Genome Walking Kit (TaKaRa Bio, Dalian, China) were used to amplify the 3' flanking sequences and 5' flanking sequences of *sul2*. The flanking sequences of *sul*2 were amplified by PCR according to the protocol of the Genome Walking Kit (TaKaRa Bio). PCR products were cloned into the pMD19-T vector and sequenced.

### DNA sequencing and analysis

The nucleotide sequences of gene cassettes embedded in class 1 integrons were sequenced on both strands by the dideoxy chain termination method using an ABI 3130 DNA Sequencer (Applied Biosystem, Foster City, California, USA). Sequence analysis was performed with DNA Sequencing Analysis Software v5.1 (Applied Biosystems). Sequence alignment was conducted using the Nucleotide BLAST program (http://www.ncbi.nlm.nih.gov/nucleotide/).

### Nucleotide sequence accession number

**Fig 1:**
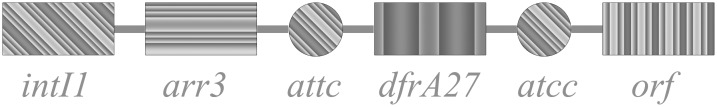
Schematic diagram of resistance gene cassettes located in the class 1 integron from Stenotrophomonas maltophilia isolates.

The sequences of the novel gene cassettes (*aar-3*-*dfrA27*, Fig 1) embedded in class 1 integron were deposited in GenBank and assigned the accession number: KC748137.

### Statistical analyses

Multiple logistic regression analyses and chi-squared tests focused on the association of SXT resistance phenotype and resistance determinants using SPSS software, 20.0 (SPSS Inc., Chicago, IL, USA). A two-sided *p*-value < 0.05 was considered to be statistically significant.

## Results

### Antimicrobial susceptibility

An alarming trend of decreased susceptibility to SXT was found over the 10-year period in clinical *S*. *maltophilia* isolates (Tables 2 or 3). MIC50 (MIC for 50% of the isolates) was also increasing during the 10-year period. According to the comparison of resistance rates between the periods of 2005 to 2009 and 2010 to 2014, the percentage of isolates resistant to SXT were significantly changed: from 29.7% in the period 2005 to 2009 to 47.1% in the period 2010 to 2014 (*p* = 0.02).

**Table 2:** Distribution of resistance determinants to trimethoprim/sulfamethoxazole resistance in clinical *Stenotrophomonas maltophilia* isolates.

year (n)	genes								SXT		MIC(mg/L)		
	*qacEΔ1-sul1(%)*	*sul2(%)*	*dfra1(%)*	*dfra5(%)*	*dfra12(%)*	*dfra17(%)*	*dfra27(%)*	*int1(%)*	S(%)	R(%)	range	MIC50	MIC90
2005(26)	11(42.3)				2(7.7)	1(3.8)		11(42.3)	21(80.8)	5(19.2)	0.25/4.75-32/608	0.25/4.75	8/152
2006(26)	12(46.2)	2(7.7)			1(3.8)	2(7.7)		13(50)	18(69.2)	8(30.8)	0.25/4.75-32/608	0.25/4.75	16/304
2007(26)	18(69.2)	3(11.5)	1(3.8)		2(7.7)	2(7.7)		17(65.4)	17(65.4)	9(34.6)	0.25/4.75-32/608	0.25/4.75	16/304
2008(24)	13(54.2)	6(25)			3(12.5)	3(12.5)		15(62.5)	16(66.7)	8(33.3)	0.25/4.75-32/608	0.25/4.75	32/608
2009(43)	23(53.5)	5(11.6)			5(11.6)			26(60.5)	30(69.8)	13(30.2)	0.25/4.75-32/608	0.5/9.5	32/608
2010(43)	23(53.5)	15(34.9)		2(4.7)	3(7.0)	2(4.7)		35(81.4)	24(55.8)	19(44.2)	0.25/4.75-32/608	0.5/9.5	32/608
2011(20)	12(60)	10(50)	1(5)	1(5)	4(20)	2(10)	3(15)	17(85)	12(60)	8(40)	0.25/4.75-32/608	1/19	32/608
2012(32)	17(53.1)	16(50)	1(3.1)	1(3.1)	4(12.5)	5(15.6)	4(12.5)	30(93.8)	15(46.9)	17(53.1)	0.25/4.75-32/608	4/76	32/608
2013(28)	25(89.3)	13(46.4)	1(3.6)		6(21.4)	5(17.9)	1(3.6)	24(85.7)	14(50)	14(50)	0.25/4.75-32/608	4/76	32/608
2014(32)	25(78.1)	7(21.9)			5(15.6)	3(9.4)		30(93.7)	17(53.1)	15(46.9)	0.25/4.75-32/608	4/76	32/608
Total (300)	179(59.7)	77(25.7)	4(1.3)	4(1.3)	35(11.7)	25(8.3)	8(2.7)	218(72.7)	184(61.4)	116(38.7)	0.25/4.75-32/608	0.5/9.5	32/608

SXT, trimethoprim-sulfamethoxazole; S, susceptible; R, resistant; MIC, Minimal inhibitory concentration; MIC50, the concentration needed to inhibit 50% of the strains; MIC90, the concentration needed to inhibit 90% of the strains

**Table 3:** Resistance determinants contributing to trimethoprim/sulfamethoxazole resistance in clinical *Stenotrophomonas maltophilia* isolates.

Genes	SXT-resistant isolates (%)	SXT-susceptible isolates (%)	Multivariate Analysis		
	n = 116	n = 184	OR	95%CI	*p*
*qacEΔ1-sul1*	94(81.0)	85(46.2)	22	(2.08–4.24)	0.000
*sul2*	59(50.9)	18(9.8)	49	(2.87–5.12)	0.000
*dfra1*	2(1.7)	2(1.1)	1	(-2.05–2.53)	0.830
*dfra5*	3(2.6)	1(0.5)	11	(-1.17–6.09)	0.180
*dfra12*	29(25)	6(3.3)	20	(1.84–4.20)	0.000
*dfra17*	18(15.5)	7(3.8)	10	(1.10–3.53)	0.000
*dfra27*	5(4.3)	3(1.6)	11	(0.49–4.33)	0.010
*int1*	97(83.6)	121(65.8)	4	(0.70–2.46)	0.000

SXT, trimethoprim-sulfamethoxazole.

### Prevalence of resistance genes

In the present study, the genes for *qacEΔ1-sul1*, *sul2* and class 1 integrase (*intI1*) were detected in 179 (59.7%), 77 (25.7%) and 218 (72.7) of the 300 *S*. *maltophilia* isolates, and the positive rates of the three genes increased gradually with the development of SXT resistance (Table 2). No class 2 or 3 integrons were found. The *dfrA* genes were detected in 76 isolates (*dfrA1*, 4 isolate; *dfrA5*, 4 isolate; *dfrA12*, 35 isolates; *dfrA17*, 25 isolates; *dfrA27*, 8 isolates) (Table 2). In addition, 85(46.2%), 18(9.8%), 121(65.8%), 2(1.1%), 1(0.5%), 6(3.3%), 7(3.8%), and 3(1.6) SXT-susceptible isolates were detected to contain the *qacEΔ1-sul1*, *sul2*, *intI1*, *dfrA1*, *dfrA5*, *dfrA12*, *dfrA17*, and *dfrA27* genes, respectively. Considering all the resistance determinants contributing to SXT resistance between the two groups of SXT-resistant isolates and SXT-susceptible isolates, the results of multiple logistic regression analysis indicated that *qacEΔ1-sul1*(OR: 22, 95% CI: 2.08–4.24, *p* = 0.000), *sul2*(OR: 49, 95% CI: 2.87–5.12, *p* = 0.000), *intI1*(OR: 4, 95% CI: 0.70–2.46, *p* = 0.000), *dfrA12*(OR: 20, 95% CI: 1.84–4.20, *p* = 0.000), *dfrA17* (OR: 10, 95% CI: 1.10–3.53, *p* = 0.000)and *dfrA27* (OR: 11, 95% CI: 0.49–4.33, *p* = 0.01) genes were significantly more prevalent in SXT-resistant isolates than SXT-susceptible isolates except *dfrA1*(*p* = 0.83) and *dfrA5*(*p* = 0.18) (Table 3).

Among the 77 *sul2*-positive isolates, according to the results of TAIL-PCR and sequence analysis, the *sul2* gene was detected to be associated with a transposase gene in 50 SXT- resistant isolates and 7 SXT- susceptible isolates.

### Detection of resistance gene cassettes in class 1 integron

Of the 218 class 1 integrase-positive isolates, 74.8% (163 isolates) were shown to contain resistance gene cassettes, which were found in 87% (101 isolates) of the SXT-resistant isolates and 33.7% (62 isolates) of the SXT-susceptible isolates. The positive rate of resistance gene cassettes in SXT-resistant isolates was significantly higher than that in SXT-susceptible isolates by chi-squared tests (*p* = 0.000). The gene cassettes in the 163 *S*. *maltophilia* isolates included those resistant to aminoglycosides, trimethoprim, β-lactams, rifampicin and chloramphenicol: *aar-3*-*dfrA27* (8 isolates), *dfrA17*–*aadA5* (25 isolates), *dfrA12*–*aadA2* (35 isolates), *aacA4*–*catB8*–*aadA1*(8 isolates), *aadB*–*aac(6****′****)-II*–*bla*_CARB-8_ (8 isolates), *arr-3-aacA4* (11 isolates), *aadB–aadA4* (9 isolates), *aacA4* (15 isolates), *aadA5*(12 isolates), *aadA1*(10 isolates), *aac(6****′****)-II*–*bla*_*CARB-8*_ (7 isolates) and *cmlA1*(15 isolates), The complete sequence of gene was deposited in the Genebank (S1 Data).

## Discussion

Due to its natural resistance to multiple classes of antimicrobial agents in *S*. *maltophilia* isolates, SXT is recommended as the first-line therapy for *S*. *maltophilia* infection. However, the treatment of *S*. *maltophilia* infections is being threatened due to the recent development of resistance to SXT. Reports from different countries have demonstrated 4%-84% resistance to SXT in *S*. *maltophilia* isolates [12–16]. In the present study, our data demonstrated a growing frequency of isolates resistant to SXT over the 10-year period. SXT-resistant isolates made up 19% of the isolates in 2005, increased to 30% in the period 2006 to 2009, and then increased to 40% from 2010 to 2014. There are statistically significant differences in SXT-resistant rates between the 2005 to 2009 period, and the 2010 to 2014 period (*p* = 0.02).

SXT resistance in *S*. *maltophilia* has been reported to be associated with the *qacEΔ1-sul1* and *sul2* genes encoding the dihydrofolate synthetase enzyme which confer resistance to sulfamethoxazole [6, 16–17]. We have found that *qacEΔ1-sul1* in combination with *sul2* confer high-level resistance to SXT in previous research [6]. However, as the study progressed, some SXT-susceptible isolates were detected to contain both *qacEΔ1-sul1* and *sul2* genes. In this research, among the 300 isolates collected during the 10-year period, the *qacEΔ1-sul1* genes were detected in 81% (n = 94) isolates that were SXT-resistant. However, 46.2% of the SXT-susceptible isolates were found to contain *qacEΔ1-sul1* genes, which may imply that resistance to SXT is caused by multiple drug resistance genes confirmed by multiple logistic regression analysis. Because resistance to SXT is coselected with resistance to quaternary ammonium compound (QAC), the presence of the QAC resistance gene *qacEΔ1* has been associated with SXT resistance [18–19]. Therefore, the use of disinfectants containing QACs might increase the risk of SXT resistance in hospital settings and natural environments.

Class 1 integrase genes were detected in 83.6% (97 isolates) of SXT-resistant isolates and 65.8% (121 isolates) of SXT-susceptible isolates; however, more resistance genes were embedded in SXT-resistant isolates. Typically, a class 1 integron structure contains *IntI1* on the 5’-conserved segment (5’CS) and *qacEΔ1-sul1* on the 3’-conserved segment. In this study, thirteen isolates were detected to contain *qacEΔ1-sul1* genes without *intI1*, and the *qacEΔ1-sul1* gene was found absent in 52 *intI1*-positive isolates, which is in agreement with previous findings on the unusual structures of the 3’- conserved region of class 1 integrons [20].

The *sul2* genes were found in 50.9% (59 isolates) of SXT-resistant isolates and 9.8% (18 isolates) of SXT-susceptible isolates, and *sul2* genes in 50 SXT- resistant isolates and 7 SXT- susceptible isolates were detected to be associated with transposase gene. The presence of *sul2* gene has increased SXT resistance, when it is associated with transposons, the gene could be further disseminated among bacteria through horizontal gene transfer in *S*. *maltophilia* isolates.

49.1% (57 isolates) of SXT-resistant isolates and 10.3% (19 isolates) of SXT-susceptible isolates were positive for *dfrA* genes encoding the dihydrofolate reductase enzyme, which confer resistance to trimethoprim. Of the *dfrA* gene subtypes, *dfrA12* and *dfrA17* were more prevalent than other genes. Besides, *dfrA12*, *dfrA17*, and *dfrA27* were found to be embedded in class 1 integron, which may imply the wide and rapid dissemination of these genes in *S*. *maltophilia* isolates in this region.

Among the 300 isolates, multiple logistic regression analysis was used in determining the contribution of resistance determinants to SXT resistance against *S*. *maltophilia* isolates among the *qacEΔ1-sul*, *dfra* and *intI1* genes. The resistance determinants including *qacEΔ1-sul1*, *sul2*, *intI1*, *dfrA12*, *dfrA17*, and *dfrA27*, but not *dfrA1* and *dfrA5*, were found to be significantly more frequent in the SXT-resistant isolates than in the SXT-susceptible isolates (*p* < 0.05). This might be attributed to fewer *dfrA1*- positive and *dfrA5*—positive strains. The difference in the prevalence of the two genes between SXT- resistant isolates and SXT-susceptible isolates did not reveal any statistical significance.

Furthermore, among 218 *intI1*-positive isolates, a total of 12 kinds of resistance gene cassettes were detected in 163 (74.8%) of *S*. *maltophilia* isolates. The positive rate of resistance gene cassettes was significantly higher in SXT-resistant isolates (87%, 101 isolates) than that in SXT-susceptible isolates (33.7%, 62 isolates) (*p* = 0.000). The most common gene cassettes located in the class 1 integrons of *S*. *maltophilia* isolates in this region were *aar-3*-*dfrA27*, *dfrA12*–*aadA2*, *dfrA17*–*aadA5*, *cmlA1*, *aacA4*, *aadA5*, *arr-3-aacA4*, *aadA1*, *aadB–aadA4*, *aacA4*–*catB8*–*aadA1*, *aadB*–*aac(6****′****)-II*–*bla*_CARB-8,_ and *aac(6****′****)-II*–*bla*_*CARB-8*_. Among the resistance gene cassettes in this research, a novel finding was the discovery of *aar-3*-*dfrA27* genes which confer resistance to rifampin and trimethoprim. As far as we know, this is the first report of the *dfrA27* gene being embedded in class 1 integron in *S*. *maltophilia* isolates, following the discovery of the *dfrA17* and *dfrA12* gene cassettes in clinical *S*. *maltophilia* isolates [6]. In addition, the prevalence of *intI1* gene and resistance gene cassettes contained in class 1 integrons have increased year by year since the development of SXT resistance, to which clinicians may need pay more attention.

In conclusion, the findings of the current study revealed an increasing presence of *qacEΔ-sul1*, *sul2*, *dfra12*, *dfra17* and *intI1* in clinical *S*. *maltophilia* isolates in China during a 10-year period. The present findings also indicated a higher prevalence of these resistance determinants in SXT-resistant isolates than in SXT-susceptible isolates. Moreover, the *qacEΔ1-sul1*, *dfrA12*, *dfrA17*, and *dfrA27* genes located in class 1 integron, and the *sul2* gene linked to transposase gene may imply the wide and rapid dissemination of resistance genes in bacteria in this region. In our opinion, this is a serious medical problem which should be of concern.

## Supporting Information

S1 Data (DOCX)
