# Supplementary material for: Increase in the Prevalence of Resistance Determinants to Trimethoprim/Sulfamethoxazole in Clinical Stenotrophomonas maltophilia Isolates in China
Source: PLoS One. 2016 Jun 16;11(6):e0157693. doi: 10.1371/journal.pone.0157693 (PMC4911037; doi:10.1371/journal.pone.0157693)
Supplement: S1 Data — (DOCX) [file pone.0157693.s001.docx]

The complete sequence of *dfrA17*–*aadA5* founded in this study was deposited in the Genebank at the link “[http://www.ncbi.nlm.nih.gov/nuccore/](http://www.ncbi.nlm.nih.gov/nuccore/GQ924479)”.

The complete sequence of *dfrA12*–*aadA2* founded in this study was deposited in the Genebank at the link “http://www.ncbi.nlm.nih.gov/nuccore/”.

The complete sequence of *aacA4*–*catB8*–*aadA1* founded in this study was deposited in the Genebank at the link “http://www.ncbi.nlm.nih.gov/nuccore”.

The complete sequence of *aadB*–*aac(6****′****)-II*–*bla*_CARB-8_ founded in this study was deposited in the Genebank at the link “http://www.ncbi.nlm.nih.gov/nuccore/”.

The complete sequence of *arr-3-aacA4* founded in this study was deposited in the Genebank at the link “http://www.ncbi.nlm.nih.gov/nuccore”.

The complete sequence of *aar-3*-*dfrA27* founded in this study was deposited in the Genebank at the link “http://www.ncbi.nlm.nih.gov/nuccore”.
